# Stress Signal Network between Hypoxia and ER Stress in Chronic Kidney Disease

**DOI:** 10.3389/fphys.2017.00074

**Published:** 2017-02-08

**Authors:** Hiroshi Maekawa, Reiko Inagi

**Affiliations:** ^1^Division of Nephrology and Endocrinology, University of Tokyo Graduate School of MedicineTokyo, Japan; ^2^Division of Chronic Kidney Disease Pathophysiology, University of Tokyo Graduate School of MedicineTokyo, Japan

**Keywords:** hypoxia, er stress, chronic kidney disease, stress signal network, UPR signaling pathways

## Abstract

Chronic kidney disease (CKD) is characterized by an irreversible decrease in kidney function and induction of various metabolic dysfunctions. Accumulated findings reveal that chronic hypoxic stress and endoplasmic reticulum (ER) stress are involved in a range of pathogenic conditions, including the progression of CKD. Because of the presence of an arteriovenous oxygen shunt, the kidney is thought to be susceptible to hypoxia. Chronic kidney hypoxia is induced by a number of pathogenic conditions, including renal ischemia, reduced peritubular capillary, and tubulointerstitial fibrosis. The ER is an organelle which helps maintain the quality of proteins through the unfolded protein response (UPR) pathway, and ER dysfunction associated with maladaptive UPR activation is named ER stress. ER stress is reported to be related to some of the effects of pathogenesis in kidney, particularly in the podocyte slit diaphragm and tubulointerstitium. Furthermore, chronic hypoxia mediates ER stress in blood vessel endothelial cells and tubulointerstitium via several mechanisms, including oxidative stress, epigenetic alteration, lipid metabolism, and the AKT pathway. In summary, a growing consensus considers that these stresses interact via complicated stress signal networks, which leads to the exacerbation of CKD (Figure 1). This stress signal network might be a target for interventions aimed at ameliorating CKD.

## Introduction

CKD is a global public health problem which has substantial impact on morbidity, mortality, and health resource utilization. The progression of CKD is defined as a decrease in glomerular filtration rate regardless of primary disease. CKD is related to a variety of metabolic abnormalities including acidosis, hypertension, anemia, and mineral bone disease (Collister et al., 2016). Chronic hypoxia of the tubulointerstitium is the common pathway that leads to end stage renal disease (Mimura and Nangaku, 2010). Hypoxia also triggers ER stress, which further contributes to the progression of CKD (Inagi et al., 2014). In this review article, we summarize the crosstalk between hypoxia and ER stress in CKD and explore possible targets for intervention.

**Figure 1:**
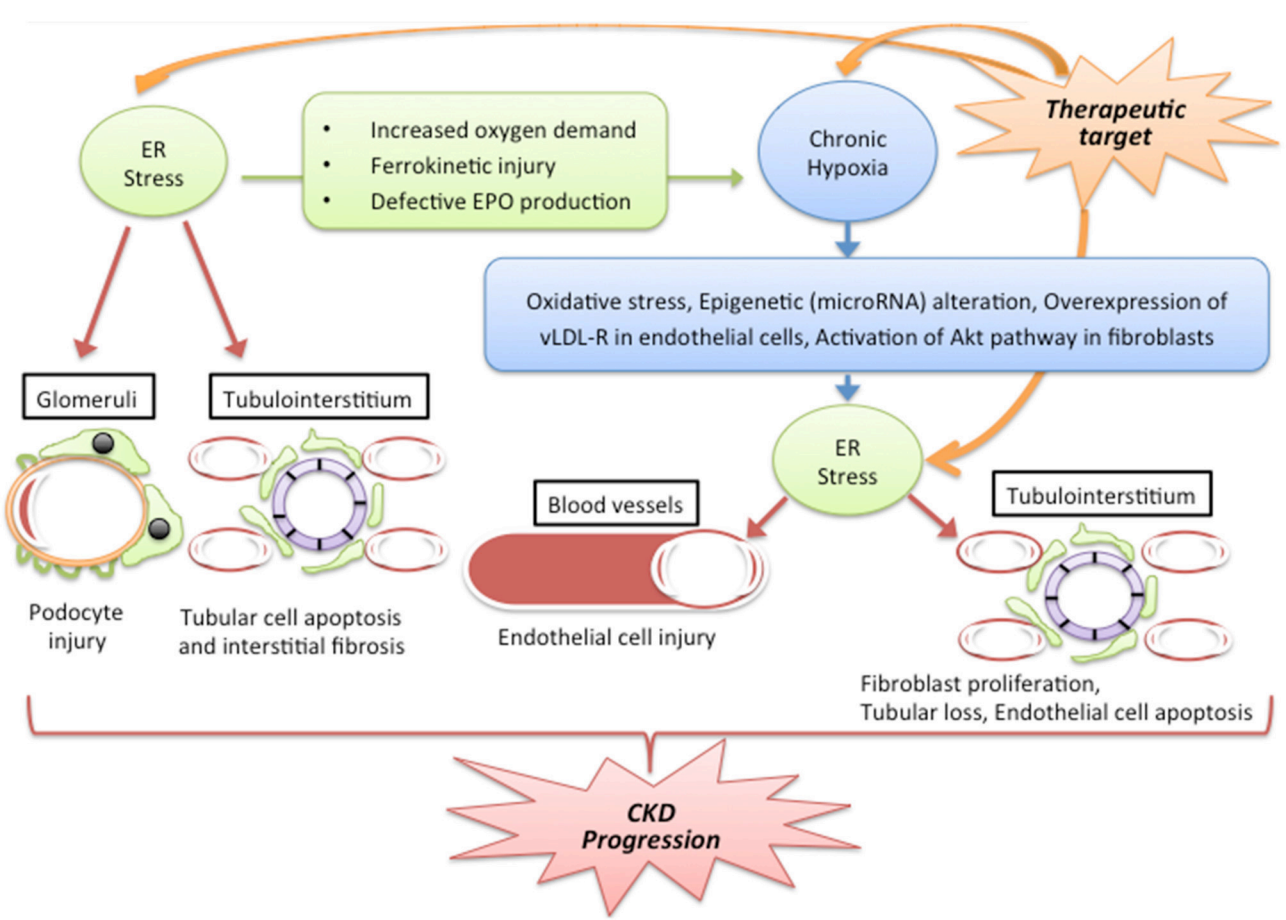
**Putative stress signal network between ER stress and hypoxia in CKD**. Abbreviations: Epo, erythropoietin; ER, endoplasmic reticulum CKD: chronic kidney disease; vLDL-R, very low lipoprotein receptor. Hypoxia and ER stress interact through a number of complicated pathways and lead to the exacerbation of CKD. The progression of CKD is caused via vascular damage, glomerular damage and tubulointerstitial injury. The mechanisms by which ER stress induces hypoxia include a change of oxygen demand in tissue, dysfunction of iron metabolism and reduction in EPO production. By contrast, chronic hypoxia induces ER stress through oxidative stress, epigenetic regulation by microRNA, overexpression of vLDL-R and the Akt pathway. These pathogenic factors could be targets for CKD therapy.

## Pathophysiology of hypoxia and ER stress in kidney disease

### Physiological hypoxia in kidney

Hypoxia is a pathologic condition which is characterized by an insufficient supply of oxygen to meet demand. The blood supply to the kidneys is very large, accounting for roughly 25% of cardiac output. However, owing to the presence of an arteriovenous oxygen shunt in the kidney (Schurek et al., 1990; Welch et al., 2001), no more than 10% of the oxygen delivered through the renal artery is utilized (Evans et al., 2008). Oxygen utilization by the kidney therefore appears to be inefficient, suggesting in turn that the kidney might be particularly susceptible to hypoxia.

#### How kidneys survive the hypoxic state

When the kidney is exposed to hypoxia, the expression of some genes changes. The master regulator of the adaptation to hypoxia is hypoxia inducible factor (HIF), a transcription factor. HIF is composed of an α-subunit (HIF-1α,2α,3α) and β-subunit [HIF-1β/AhR nuclear translocator (ARNT)]. Although HIF-1β is constitutively expressed, HIF-α members are degraded in normoxic conditions. HIF-α is hydroxylated by a prolyl hydroxylase domain-containing protein (PHD), and the binding of HIF–α protein to the von Hippel Lindau protein (pVHL) results in ubiquitination and degradation. Under hypoxia, HIF-α escapes this degradation and dimerizes with HIF-1β. The dimer translocates into the nucleus and binds to the hypoxia-response element (HRE) of HIF-target genes. This results in the activation of target genes involved in angiogenesis, erythropoiesis, and glycolysis (Mimura and Nangaku, 2010; Shoji et al., 2014).

#### Pathogenic hypoxia in the kidney

Various pathogenic conditions induce chronic kidney hypoxia, including hypertension and diabetes. Some studies have shown that following renal ischemia, density of the peritubular capillaries decreases, as does oxygen tension in the kidney (Basile et al., 2001, 2003). Furthermore, the systemic hemodynamic changes and vasoconstriction associated with the renin-angiotensin system result in a decrease in peritubular capillary flow (Korner et al., 1994). Hypoxia might also be induced via tubulointerstitium fibrosis, in which the distance between the capillary and tubular cells is extended, leading to further impairment of oxygen delivery (Norman and Fine, 2006). In addition, some conditions increase oxygen demand, including glomerular hyperfiltration induced by hyperglycemia, (Korner et al., 1994), and anemia decreases oxygen delivery to the kidney (Johannes et al., 2007).

### ER stress and its stress signal UPR pathway

The ER is the main organelle involved in the quality control of proteins. This process, termed proteostasis, involves protein synthesis, protein folding, and the degradation of malfolded proteins. The quality of proteins is maintained through the UPR pathway, an integrated pathway which transduces information about protein folding conditions from the ER to the nucleus by controlling the activity of specific downstream transcription factors (Inagi et al., 2014; Rivas et al., 2015). The adaptive UPR pathway is regulated by the three major pathway transducers present in the ER lumen, named inositol-requiring protein 1 (IRE1), pancreatic eukaryotic translation initiation factor 2α (eIF2α) kinase (PERK) and activating transcription factor 6 (ATF6). These transducers are kept in an inactive state in normal conditions via binding to the ER chaperon GRP78 (78 kD glucose-regulated protein). However, the maintenance of protein metabolism is disturbed under various pathogenic stresses, including hypoxia. This disturbance results in the accumulation of unfolded proteins through ER dysfunction, which in turn causes cell damage. This series of events is termed ER stress. Under ER stress, GRP78 is dissociated from transducers and binds to unfolded proteins. The free transducers are thus activated, causing the UPR transcription factors, including X-box-binding protein 1 (XBP1) and ATF4, to translocate into the nucleus. This translation leads to up-regulation of the expression of UPR-target genes involved in protein folding and ER-associated protein degradation (ERAD). Activated ATF6 translocates into the Golgi apparatus and is cleaved to form ATFp50, a transcription factor which activates the expression of the UPR-target gene related to ERAD. However, if the adaptive UPR pathway is overcome by chronic or severe ER stress and proteostasis cannot be maintained, the predominant outcome is the induction of ER stress-related apoptosis, which occurs via the activation of C/EBP homologous protein (CHOP) (Kimata et al., 2004; Inagi, 2010; Pincus et al., 2010). ER stress is associated with many diseases, including diabetes (Rivas et al., 2015), chronic heart failure (Cominacini et al., 2015) and some neurodegenerative diseases (Soto, 2003). Moreover, many recent studies have reported a relationship between the UPR pathway and glomerular and tubular cell damage in various kidney diseases (Inagi et al., 2014).

#### Pathophysiology of ER stress in kidney disease

Many studies have revealed the effects of the pathogenesis of the UPR pathway in the kidney. The podocyte slit diaphragm is important as a glomerular filtration barrier. The structural components of the slit diaphragm [nephrin, alpha-actinin-4, and CD2-associated protein (CD2AP)] are subject to a mutation which causes defective protein folding in the ER of podocytes. The accumulation of malfolded proteins of the slit diaphragm induces structural and functional damage associated with ER stress and subsequent proteinuria (Cybulsky et al., 2009; Chiang and Inagi, 2010; He et al., 2011). Various pathogenic factors also trigger ER stress in podocytes, including complement complex (Cybulsky, 2013) and calcium entry via transient receptor protein 6 (TRPC6) (Chen et al., 2011). ER stress also injures podocytes via increased expression of monocyte chemoattractant protein 1 (MCP-1), which plays a central role in the inflammation associated with diabetic nephropathy. The structural and functional properties of tubular epithelial cells are closely dependent on ER proteostasis. Exposure of tubular cells to severe or long-term stress induces UPR pathway-mediated apoptosis and leads to the progression of CKD. Well-known examples of adverse factors that induce UPR pathway-related cell apoptosis, reduce the repair capacity of tubular cells and accelerate the progression of kidney disease include proteinuria (Ohse et al., 2006), hyperglycemia (Lindenmeyer et al., 2008), uremic toxins (Kawakami et al., 2010), and nephrotoxins, including cisplatin (Ozkok and Edelstein, 2014). Furthermore, ER stress has also been demonstrated in a unilateral ureteral obstruction tubulointerstitial fibrosis rat model (Chiang et al., 2011), in which excessive UPR activation over the adaptive UPR pathway contributed to tubular cell apoptosis and the resulting fibrosis.

## Interaction of hypoxia and ER stress

### Hypoxia to ER stress

Chronic hypoxia triggers ER stress. Hypoxia induces cell stress, which leads the production of reactive oxygen species (ROS), a situation termed oxidative stress. ROS are produced in several organelles, including the ER. Altered redox homeostasis in the ER cause ER stress. These findings indicate a close link between hypoxia, oxidative stress, and ER stress (Sena and Chandel, 2012; Cao and Kaufman, 2014; Inagi et al., 2014). For example, we previously reported the epigenetic regulation of the cross-talk of hypoxia-oxidative stress-ER stress by microRNA. We found that miR-205, a microRNA which is predominantly expressed in kidney tubular cells, maintains tubular homeostasis by regulating the expression of PHD1, which negatively controls HIF1α and ATF4. HIF1α is a transcription factor of the HIF pathway and ATF4 is a transcription factor of the URP pathway, and the two act together to regulate antioxidant enzyme expression (Muratsu-Ikeda et al., 2012). Furthermore, hypoxia results in activation of the PERK/eIF2α axis of the UPR pathway by Akt (also known as protein kinase B or PKB), a serine/threonine kinase member of the AGC family of protein kinases which is involved in cell growth, proliferation, protein translation, and cell survival. Cells without Akt isoform 1 and 2 do not induce either PERK or eIF2α phosphorylation, even in hypoxic conditions, demonstrating that Akt mediates PERK/eIF2α activation during hypoxia (Blaustein et al., 2013). In addition, hypoxia triggers ER stress and induces very low density lipoprotein receptors (vLDL-R) in vessel endothelial cells. Knockdown or overexpression of vLDL-R improves or exacerbates hypoxia-induced ER stress, demonstrating that vLDL-R induces endothelial cell apoptosis via ER stress (Yang et al., 2014). Thus, these mechanisms might be targets for interventions aimed at preventing the CKD progression associated with vessel and tubulointerstitial damage.

### ER stress to hypoxia

ER stress is reported to suppress the production of erythropoietin (EPO), a hematopoietic hormone regulated by the HIF pathway. This suppression of EPO by ER stress is inversely correlated with ATF4 expression, and the binding of ATF4 to the 3′ enhancer region of EPO gene abolished the enhancer activity (Chiang et al., 2013). ER stress also changes ferrokinetics. Hepcidin is a peptide hormone secreted by the liver which controls iron homeostasis. The production of Hepcidin is mediated by inflammation and iron. The overproduction of hepcidin causes anemia, but its deficiency leads to hemochromatosis. One report revealed that ER stress also induces hepcidin expression and causes hypoferremia in mice. CREBH (cyclic AMP response element–binding protein H), an ER stress–activated transcription factor, binds to and trans-activates the hepcidin promoter. Hepcidin induction in response to exogenously administered toxins or the accumulation of unfolded proteins in the ER is defective in CREBH knockout mice, indicating a role for CREBH in ER stress–regulated hepcidin expression (Vecchi et al., 2009). These reports indicate that ER stress is related to hematopoiesis and iron metabolism, and that ER stress might therefore be related to hypoxia via the mediation of anemic conditions.

The mechanisms described above could lead to a decrease in kidney function via deteriorating hypoxia by induction of anemia. In turn, a decrease in kidney function results in the accumulation of uremic toxins. Indoxyl sulfate (IS), a representative uremic toxin, induces ER stress in cultured human proximal tubular cells, as demonstrated by an increase in CHOP, and inhibits the proliferation/repair of tubular cells (Kawakami et al., 2010). Moreover, IS also increases oxygen consumption in kidney proximal tubular cells and decreases renal oxygenation. On the other hand, IS suppresses mRNA expression of EPO, demonstrating a link between IS and hypoxia as well as ER stress (Chiang et al., 2011). In fact, IS suppresses the induction of HIF-1 target gene expression in hypoxic conditions through dysfunction of the HIF-1α C-terminal transactivation domain (CTAD). The suppression of HIF-1 activity by IS is correlated with up-regulation of CBP/p300-interacting transactivator with the Glu/Asp-rich carboxy-terminal domain 2 (CITED2), which is a negative regulator of HIF-1 activity. Namely, IS increases CITED2 expression via post-transcriptional mRNA stabilization (Tanaka et al., 2013). Asai et al have shown that IS suppresses HIF activation, and subsequent EPO production via AhR activation, and that AhR blockade improves IS-induced suppression of HIF activation (Asai et al., 2016). These findings suggest that IS may be a pathogen that perturbs cross-talk between the UPR pathway of ER stress and the HIF pathway of hypoxia. Taken together, these findings indicate that the removal of IS, blockage of AhR, inhibition of hepcidin production and mediation of the UPR pathway, including ATF4, might be therapeutic targets in ameliorating hypoxia and subsequent CKD progression.

## Conclusion

Hypoxia and ER stress act together to induce a deterioration in kidney function. This adverse effect is mediated by the formation of a complicated signal network between them. Furthermore, uremic toxins such as IS also induce ER stress and subsequent hypoxia through suppression of erythropoiesis and the exacerbation of tubular fibrosis. These pathogenic factors could be targets for CKD therapy.

## Author contributions

This manuscript was written by HM and edited by RI.

## Funding

This work was supported by the Japan Society for the Promotion of Science Grants-in-Aid for Scientific Research (25461207, 15KT0088, and 16K15465 to RI), Yakult Bio-Science Foundation (to RI), and a research grant from Kyowa Hakko Kirin Co., Ltd (to RI).

### Conflict of interest statement

The authors declare that the research was conducted in the absence of any commercial or financial relationships that could be construed as a potential conflict of interest.
